# Distinct effects of endosomal escape and inhibition of endosomal trafficking on gene delivery via electrotransfection

**DOI:** 10.1371/journal.pone.0171699

**Published:** 2017-02-09

**Authors:** Lisa D. Cervia, Chun-Chi Chang, Liangli Wang, Fan Yuan

**Affiliations:** Department of Biomedical Engineering, Duke University, Durham, North Carolina, United States of America; Universidad de Castilla-La Mancha, SPAIN

## Abstract

A recent theory suggests that endocytosis is involved in uptake and intracellular transport of electrotransfected plasmid DNA (pDNA). The goal of the current study was to understand if approaches used previously to improve endocytosis of gene delivery vectors could be applied to enhancing electrotransfection efficiency (eTE). Results from the study showed that photochemically induced endosomal escape, which could increase poly-L-lysine (PLL)-mediated gene delivery, decreased eTE. The decrease could not be blocked by treatment of cells with endonuclease inhibitors (aurintricarboxylic acid and zinc ion) or antioxidants (L-glutamine and ascorbic acid). Chemical treatment of cells with an endosomal trafficking inhibitor that blocks endosome progression, bafilomycin A1, resulted in a significant decrease in eTE. However, treatment of cells with lysosomotropic agents (chloroquine and ammonium chloride) had little effects on eTE. These data suggested that endosomes played important roles in protecting and intracellular trafficking of electrotransfected pDNA.

## Introduction

Electrotransfection is a non-viral method for gene delivery. It facilitates delivery of naked plasmid DNA (pDNA) into cells through application of a pulsed electric field [1]. Although it was first reported by Neumann *et al*. in 1982 [2], mechanisms of how the DNA is delivered from extracellular space to cell nucleus for successful gene expression remain largely unknown. Two theories have been proposed for electrotransfection. The traditional theory, commonly referred to as the pore theory, states that application of pulsed electric field creates transient, hydrophilic pores in the plasma membrane, in a process known as electroporation, that permit pDNA to enter the cell [3]. Currently, there are no direct experimental measurements of the pore size and its dynamics. Results from numerical simulations have predicted that the life-time of pores, which are larger than the size of pDNA, is on the order of 10 msec [4,5]. This time scale is several orders of magnitude shorter than the time frame of pDNA uptake, which has been observed to be on the order of 10 min [6,7]. Thus, the prolonged uptake of pDNA cannot be explained by the pore theory. Recently, a few studies investigated endocytic pathways involved in electric pulse-mediated internalization of pDNA, based on treatments of cells with endocytic inhibitors [7–10]. These studies have resulted in a new theory, suggesting that multiple endocytic pathways are involved for pDNA to enter the cytoplasm in electrotransfection.

It is widely accepted that efficiencies of virus- or cationic carrier-mediated gene delivery depend on the ability of genes to escape from early endosomes to avoid being degraded enzymatically in lysosomes [11]. Some gene delivery methods naturally induce endosomal escape, such as adeno-associated virus (AAV) [12]. For those methods that do not have such a capability, endosomal escape can be achieved by using various endosome-disruptive approaches. One approach is to treat cells with small chemicals, such as Ca^2+^ [13], chloroquine [14], and sucrose [15]. Ca^2+^ may induce endosomal escape via the proton sponge effect [16], which works by causing osmotic swelling and eventual rupture of the vesicles [11,17–20]. Similarly, chloroquine can induce the vesicle rupture through protonation in acidic environment of late endosomes and lysosomes [11]. Sucrose causes the vesicle rupture via a different mechanism. In mammalian cells, endocytosed sucrose often accumulates in endosomes and lysosomes because of the lack of intracellular disaccharidase for metabolizing sucrose [15]. The sucrose accumulation can induce osmotic swelling of the vesicles.

A second approach is to deliver pDNA with carriers containing materials with endosome-disruptive properties [17,21–25]. It has been observed that some cationic polymers, such as polyethylenimine (PEI) and polyamidoamine (PAMAM), can induce endosomal escape through the proton sponge effect [17,18,25,26]. However, other polymeric carriers, such as poly-L-lysine (PLL), do not have pH buffering properties and therefore are unable to induce endosomal escape by themselves [27]. To improve the PLL buffering capacity, functional moieties, such as histidine, are frequently included in the polymeric system so that the proton sponge effect can occur [25,28,29]. The cationic-lipid based methods, such as lipofectamine 2000, utilize a unique, flip-flop mechanism to induce endosomal escape. Due to electrostatic interactions, anionic phospholipids in the endosomal membrane leaflet facing cytoplasm can translocate to the inner leaflet, diffuse laterally, and form charge-neutralized ion pairs with the cationic lipoplexes. The process may destabilize the membrane and displace the nucleic acids from the lipoplexes, thereby releasing them into the cytosol [24,25,30,31].

A third approach is called photochemical internalization (PCI) that targets specific membranes with a photosensitizer. Upon exposure to light with a specific wavelength, the photosensitizer reacts with photons to generate reactive oxygen species (ROS), which can cause endosomal membrane damage [32]. The damage is confined within a radius of 10-20 nm from the photosensitizer location because ROS has a short half-life (~20 nsec) [33]. Endosomal escape of macromolecules can happen within a few seconds after PCI when endosome membranes are ruptured photochemically [34,35].

Although endosomal escape is considered to be crucial for gene delivery, an important question is, when is the optimal time for the escape to occur? Different pathways for gene trafficking in the cytoplasm may commonly involve transition from early endosome to endosomal carrier vesicle (ECV) or multivesicular body (MVB), to late endosome, and to lysosome [36]. The release of pDNA from endosomes avoids degradation in lysosomes and can potentially increase efficiency of gene delivery. Indeed, data in the literature have shown that transfection efficiency can be increased when the gene is released from early endosomes immediately after cell transfection with PEI or PLL [37]. However, it is unknown if similar approaches can be applied to improve electrotransfection. Therefore, we investigated how induction of endosomal escape or inhibition of specific transitions in endocytic pathways could affect gene delivery via electrotransfection.

## Materials and methods

### Cell culture

COS7 (African green monkey kidney fibroblast-like), HEK293 (human embryonic kidney) and HCT116 (human colorectal carcinoma) cell lines were obtained from ATCC (Manassas, VA). COS7 and HEK293 cell lines were cultured in high glucose Dulbecco’s modified Eagle’s medium (Gibco, Grand Island, NY), supplemented with 10% (vol/vol) fetal bovine serum and 1% penicillin streptomycin (15140–122, Gibco). HCT116 was cultured in McCoy medium with 10% FBS and 1% penicillin streptomycin. Cells were passaged every 2–3 days and were incubated at 37°C in 5% CO_2_ and 95% air.

### Solutions of pharmacological agents

We used double-distilled water (ddH_2_O) to prepare stock solutions of all pharmacological agents with the exception of bafilomycin A1, which was dissolved in DMSO. The cells in control groups were treated with the equivalent volumes of ddH_2_O or DMSO without the addition of the pharmacological agents. The final concentrations used in treatment of cells were 2 mM, 4 mM, or 6 mM for L-glutamine (G3202, Sigma-Aldrich, St. Louis, MO), 0.5 mM or 1 mM for ascorbic acid (A4544, Sigma-Aldrich), 80 μM for zinc sulfate (Z0251, Sigma-Aldrich), 10 μM for aurintricarboxylic acid (A1895, Sigma-Aldrich), 1 μM for bafilomycin A1 (B1793, Sigma-Aldrich), 100 μM for chloroquine (C6628, Sigma-Aldrich), and 10 mM for ammonium chloride (A9434, Sigma-Aldrich).

### Labeling of early endosomes and pDNA

COS7 cells were seeded at a density of 0.5x10^6^ cells/well in a 6-well plate. On the next day, cells were transfected with a plasmid encoding Rab5-GFP (Plasmid #61802, Addgene, Cambridge, MA) via lipofectamine 2000 (11668019, Invitrogen, Carlsbad, CA) at a ratio of 1:3 (DNA:lipofectamine) for 4 hours. Then, the cells were transferred to the full medium, cultured for 24 hours, and electrotransfected (see electrotransfection protocol below). Twenty minutes after electrotransfection, the cells were treated with light for 2 min (see Photochemical Internalization section below), and fixed with 2% paraformaldehyde. The samples were then imaged with a confocal microscope (see Light Microscopy section below). The pDNA was labeled with LabelIT CX-Rhodamine (MIR3100, Mirus, Madison, WI) with a 1:5 ratio as per manufacturer’s protocol. The fluorescent dye was covalently bound to DNA and the labeled plasmid was purified by ethanol precipitation and re-suspended in ddH_2_O before use.

### Photochemical internalization

PCI was performed with an amphiphilic, sulfonated photosensitizer, TPPS_2a_ (T40637, Frontier Scientific, Logan, UT). TPPS_2a_ has a peak absorbance at 415 nm. Thus, we chose the blue light source to be 375–550 nm with a peak at 435 nm to generate ROS. There were two PCI protocols: light-after and light-before. In both protocols, cells were seeded at a density of 2x10^4^ cells/cm^2^ and allowed to attach to the surface of a 6-well plate (Clontech, Mountain View, CA) for at least 6 hours. Thereafter, TPPS_2a_ was added to the culture medium at a concentration of 0.4 μg/ml from a stock dissolved in DMSO at 2 mg/ml. After 18 hours, cells were washed three times with cell culture medium and incubated in photosensitizer-free cell culture medium for 4 hours before light treatment to remove the photosensitizer from the plasma membrane through dissociation of photosensitizer from the membrane and membrane exchange between cell surface and intracellular vesicles. As a result, the plasma membrane would not be permeabilized during light treatment, which was critical to reduce cytotoxicity of PCI. The time at which the light treatment was administered relative to the time point of transfection differed for the two PCI protocols. In the light-after protocol, cells were electrotransfected with pEGFP-N1, and then exposed to the blue light at an irradiance of 1.5 mW/cm^2^ for two minutes at 10 min post application of electric field. The light was delivered by two Osram L 18/67 bulbs. Upon light stimulation, the photosensitizer generated ROS that ruptured the endosomal membrane. It was possible that light treatment and ROS might damage naked pDNA [55]. Therefore, the alternative, light-before protocol was also applied to the study, which involved the exposure of cells to the blue light to create leaky vesicles at approximately one minute prior to electrotransfection of pDNA.

### PCI validation

To verify that PCI treatment could induce endosomal escape in our study, a dextran uptake assay was performed, in which rhodamine B labeled dextran with molecular weight of 10,000 (D1824, Invitrogen) was prepared at a final concentration of 100 μg/ml. It was added to cell culture medium, allowing cells to internalize it for 20 min at 37°C. The cells were then collected, washed, and placed on a microscope slide. Punctate distribution of dextran in cells was examined under a fluorescence microscope both before and after PCI treatment using the light-after protocol described above.

To verify that PCI treatment could increase efficiency of PLL-based gene delivery method, we prepared a stock solution of PLL (P7890, Sigma-Aldrich) with molecular weight of 15,000–30,000 by dissolving the polymer in ddH_2_O (1 mg/ml), and filtered it with a 0.2 μm filter. To create PLL-pDNA complexes, 5.3 μg of PLL was added to 75 μl ddH_2_O. This solution was dropwise added to a solution containing 5 μg of pEGFP-N1 in 75 μl ddH_2_O. These complexes were incubated at room temperature for 30 min and then used to treat cells for 4 hours at 37°C. There were three different groups in the study. In all groups, cells were pre-loaded with TPPS_2a_ (see the procedure above) before transfection. In two experimental groups, cells were exposed to the blue light for a period of 1 or 5 min immediately after transfection with PLL. In the control group, cells were not treated with the light. Transfection efficiency of PLL was quantified with flow cytometry at 24 hours post transfection.

### Electrotransfection

Each transfection was performed with 10^6^ cells. The cells were suspended in 100 μl of pulsing buffer (HeBS) containing 6 μg of pEGFP-N1 plasmid (Clontech, Palo Alto, CA) on ice. The cell solution was transferred to electroporation cuvettes with two parallel plate electrodes spaced 4 mm apart. 8 electric pulses were applied to the cuvettes at a field strength of 160 V/4 mm, 5 ms duration and 1 Hz frequency for COS7 and HEK293 cells and 6 electric pulses at a field strength of 240 V/4 mm, 5 ms duration and 1 Hz frequency for HCT116 cells using a BTX ECM 830 Square Wave Electroporation System (Harvard Apparatus, Holliston, MA). After pulsing, the cuvettes were kept at room temperature for 10 min to allow the cells to recover before pipetting them to a 6-well plate with cell culture medium. Flow cytometry was performed at 24 hours post electrotransfection.

### Light microscopy

For dextran release after PCI treatment, images of cells were acquired with a fluorescence microscope (Axiovert S100, Carl Zeiss, Thornwood, NY) equipped with an X-Cite Series 120PC (EXFO) light source and Image EMX2 EM-CCD Digital Camera (Hamamatsu). The excitation and emission filters for imaging dextran were BP550/25 and BP605/70 (Chroma, Bellows Falls, VT), respectively. For imaging early endosomes and pDNA, the Andor XD revolution spinning disk confocal microscope was used in the Light Microscopy Core Facility (LMCF) at Duke University. The lasers provided excitation light at wavelengths of 488 and 561 nm for imaging Rab5-GFP and rhodamine-labeled pDNA, respectively.

### Colocalization analysis

Colocalization of rhodamine-labeled pDNA and an early endosomal marker (Rab5-GFP) was determined by using the FIJI software with Coloc 2 plug-in. For each cell, a z-stack of confocal images were first obtained. The cell in the images was masked to exclude extracellular regions. All pixels in the z-stack from the red channel that co-localized with those from the green channel were counted. The results were used to calculate the Manders’ co-localization coefficient (M) [10], which was defined as the percent of rhodamine (i.e., pDNA) positive pixels that were also GFP (i.e., Rab5) positive. A total of 10 cells were analyzed for each group.

### Flow cytometry

The medium in the 6-well plates was aspirated and the adherent cells were washed with phosphate-buffered saline (PBS) without Ca^2+^ and Mg^2+^, trypsinized and resuspended in medium containing propidium iodide (PI) (5 μg/ml). The flow cytometer (BD FACSCanto II, Becton Dickinson, Franklin Lakes, NJ), equipped with 488 and 633 nm lasers, was used to determine the amounts of cells with GFP expression and dead cells. The flow cytometer was set to collect 10,000 events for each sample. Control cells were used to correct for autofluorescence in flow cytometry analysis, which were prepared under the same conditions as those for experimental cells, except that no plasmid was added to the transfection buffer.

FlowJo software was used for data analysis. Forward and side light scatter gating were used to exclude debris and isolate the cell population of interest. The apparent eTE was defined as the percentage of viable cells expressing GFP (PI negative, GFP positive) among the total number of viable cells (PI negative). Viability of cells after each treatment was determined by setting the flow cytometer to collect events for 20 seconds for each sample, and applying forward light scattering gating and fluorescence detection to isolate the live cell population only (i.e., PI negative). The viability was defined as the ratio of live cells in the treated group versus the non-treated control for each experiment [93,94]. The viability data were multiplied by the apparent eTE described above to obtain the actual eTE. All data of eTE in the paper refer to actual eTE.

### Statistical analysis

The mean and standard error of the mean were reported for all data. The Mann-Whitney U Test was used to statistically compare data from two different groups. The difference was considered to be significant if the P-value was <0.05.

## Results

### Enhancement of PLL-mediated gene delivery via PCI-induced endosomal escape

**Fig 1:**
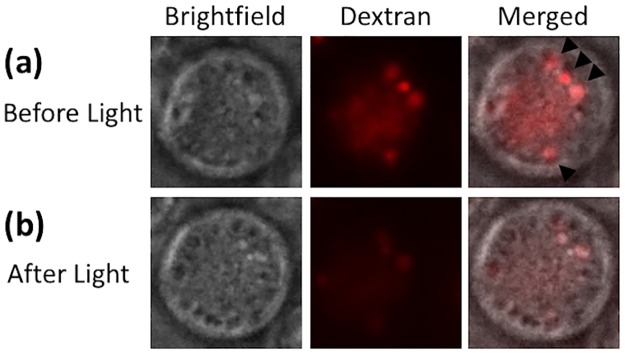
Release of rhodamine B-labeled dextran from endosomes following light treatment. Intracellular vesicles were preloaded with the photosensitizer. (a) The image of a COS7 cell before light treatment. It was taken at 20 min after the cell was incubated at 37°C with dextran (10,000 MW). The light treatment was performed immediately after the image acquisition. (b) The image of the same cell at 10 min after light treatment. The dextran was punctate within endosomes before light treatment (top panel). However, upon light treatment, dextran diffused out of endosomes, and spread in the cytosol (bottom panel).

**Fig 2:**
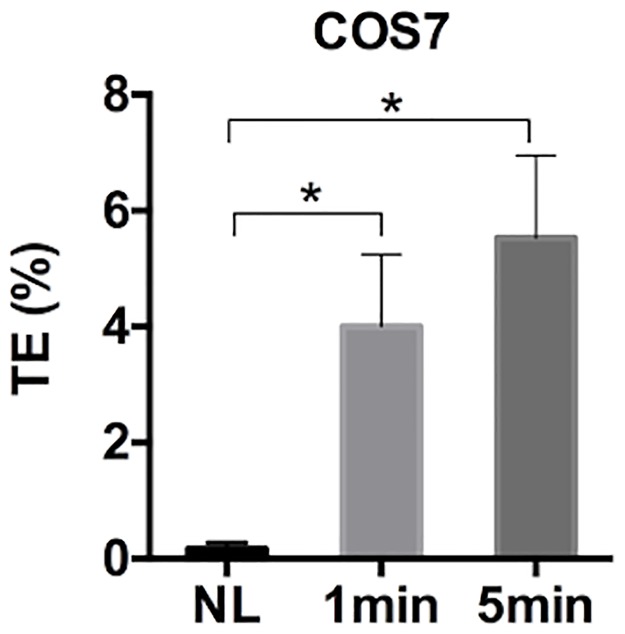
Efficiency of cell transfection with Poly-L-lysine (PLL). Intracellular vesicles were preloaded with the photosensitizer. COS7 cells were transfected with PLL-pDNA complexes for 4 hours at 37°C. Then, the cells were exposed to blue light for 0 (i.e., no light (NL) control), 1 min, and 5 min. The transfection efficiency (TE) was quantified at 24 hours post transfection. (n = 4, *P<0.05, Mann-Whitney U test).

To show that the PCI technique could facilitate endosome escape of macromolecules, we first loaded endosomal membranes with the photosensitizer and fed COS7 cells with rhodamine B-labeled dextran (10,000 MW) at 37°C. After 20 min, dextran was internalized, presumably via endocytosis [38–40], and accumulated in intracellular vesicles (Fig 1a). Then, cells were exposed to blue light. At 10 min post exposure, the punctate accumulation of dextran was significantly reduced in the cell (Fig 1b), suggesting that majority of dextran molecules had escaped from the vesicles. In a separate experiment, we observed that the PCI-induced endosomal escape could enhance efficiency of PLL-mediated transfection of the same cell line (Fig 2). Since it is known that both dextran uptake and PLL-mediated gene delivery occur via endocytosis [38–42], the data described above indicated that the PCI technique could significantly enhance gene delivery through facilitation of pDNA escape from endosomes.

### Effect of PCI-induced endosomal escape on electrotransfection

**Fig 3:**
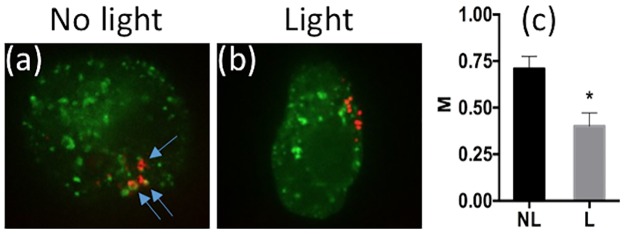
Colocalization of pDNA with Rab5. (a) Typical image of pDNA and Rab5 distributions in a COS7 cell without light treatment. It was obtained with confocal microscopy. Blue arrows indicate the points of colocalization of rhodamine-labeled pDNA (red) and Rab5-GFP (green). (b) Typical image of pDNA and Rab5 distributions in another COS7 cell after light treatment. The PCI treatment was performed with the light-after protocol, where the cells were treated with light for 2 min at 20 min after electrotransfection. Then, cells were fixed with 2% paraformaldehyde and imaged with a confocal microscope. (c) Quantitative analysis of colocalization. Manders’ colocalization coefficient (M) was calculated for each z-stack of confocal images. The light (L) treated group showed a significant decrease in the colocalization of pDNA with Rab5, compared to the no light (NL) treated group. (n = 10, *P<0.0005, Mann-Whitney U test).

To study if the PCI technique could improve electrotransfection, intracellular locations of rhodamine-labeled pDNA were first visualized under a confocal microscope, with respect to an early endosomal marker, Rab5, before and after light treatment. As expected, cells that received no light treatment showed higher colocalization of pDNA with Rab5, whereas cells in the light treated group showed less colocalization with Rab5 (Fig 3a and 3b), indicating escape of pDNA from endosomes after light treatment. The colocalization was also quantitatively characterized by the Manders’ coefficient, which decreased significantly after light treatment (Fig 3c).

**Fig 4:**
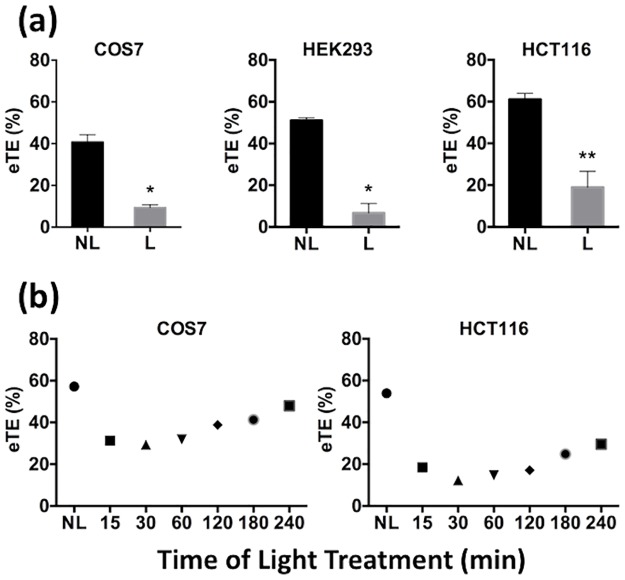
Effects of light-after treatment on eTE. (a) PCI treatment was performed with the light-after protocol. All cells were exposed to light at 10 min post application of electric field. The treatment resulted in a significant reduction in eTE in COS7, HEK293 and HCT116 cells. (n = 4, *P<0.05, **P<0.01, Mann-Whitney U test.) (b) Dependence of eTE on the time of light treatment. Cells were divided into 7 groups. Cells in each group were exposed to light at a specific time points indicated in the horizontal axis of the plots, or not treated with light (NL). The eTE was quantified at 24 hours post electrotransfection. Each symbol in Panel (b) represents the mean eTE from two repeated experiments.

Next, we used the PCI technique to trigger pDNA release from endosomes at specific time points after electrotransfection. The rationale for the experimental design was that at different time points, pDNA might localize in different endocytic compartments, such as early endosomes, ECV/MVBs, late endosomes, and lysosomes. Surprisingly, endosomal escape triggered at different time points all resulted in a decrease in electrotransfection efficiency (eTE), compared to the controls (Fig 4a and 4b). Although the efficiency appeared to recover at later time points, it never reached or exceeded the level of eTE in control cells. Thus, the recovery was likely to be due to the washout of photosensitizer from membranes in intracellular compartments.

**Fig 5:**
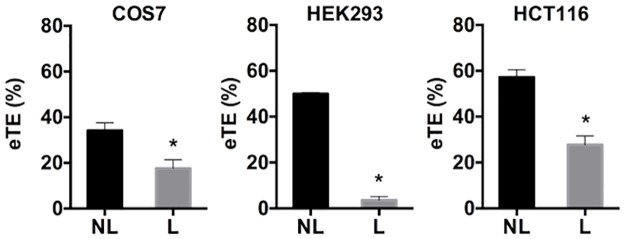
Effects of light-before treatment on eTE. PCI treatment was performed with the light-before protocol that prevented pDNA damage by the light treatment or ROS induced by photochemical reactions. However, the eTE still decreased for COS7, HEK293 and HCT116 cell lines after light (L) treatment, compared to the no light (NL) control. (n = 4, *P<0.05, Mann-Whitney U test).

The decrease in eTE could be due to oxidative damage of pDNA induced by photochemical reactions or degradation of pDNA by endonucleases upon release into the cytosol. To investigate which factors were more important, we first compared two complementary experimental protocols for PCI: light-before and light-after, which created leaky vesicles before and after transfecting the cells with pDNA, respectively. It was observed that both protocols resulted in significant decreases in eTE in all three different cell lines (COS7, HEK293, and HCT116) (Figs 4 and 5). Furthermore, the decrease in eTE could not be blocked by treatment of cells with antioxidants, including L-glutamine (at concentrations of 2, 4, and 6 mM) and ascorbic acid (0.5 mM and 1 mM). The treatments were performed for 1 hour prior to light exposure or during light exposure and continued for 4 hours after the exposure. Similar results were observed after all treatments. These data, together with the fact that half-life of ROS, generated during light treatment in the presence of photosensitizer, is short [33,43], suggested that the decrease in eTE was unlikely to be caused by ROS-induced damage in pDNA.

To investigate whether endonucleases in the cytosol could degrade pDNA upon endosomal release, cells were pre- or post-treated with endonuclease inhibitors, aurintricarboxylic acid (ATA) at 10 μM and zinc ion (Zn^2+^) at 80 μM. The period of treatment was either 10 min or 1 hour. Additionally, we tried applying these endonuclease inhibitor treatments immediately after pulsing for a period of 24 hours as well as including the inhibitors in the pulsing buffer. Previous studies have shown that these inhibitors prevent degradation of pDNA [44–46]. In the current study, however, both pre- and post-treatments of cells with ZnSO_4_ or ATA failed to improve eTE after PCI-induced endosomal escape of pDNA (data not shown).

### Inhibition of intracellular trafficking with pharmacological agents

**Fig 6:**
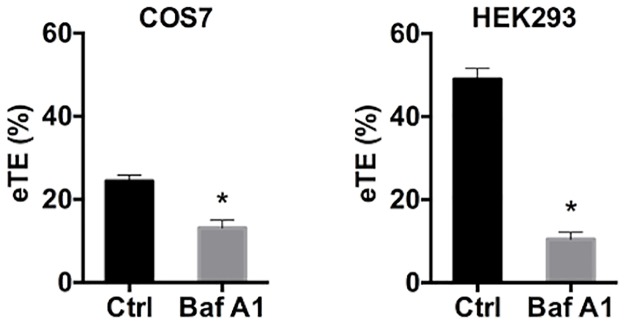
Effects of bafilomycin A1 pretreatment on transgene expression. COS7 and HEK293 cells were treated with either buffer in the control (Ctrl) or an endocytic trafficking inhibitor, bafilomycin A1 (Baf A1), at 1 μM for 1 hour prior to electrotransfection; and the eTE was quantified at 24 hours post electrotransfection. (n = 4, *P<0.05, Mann-Whitney U test).

To further investigate whether there existed specific stages in intracellular pathways that were critical for pDNA transport in electrotransfection, we treated cells with specific endosome acidification-interfering molecules [47], which could interfere with intracellular trafficking at different stages of endosomal maturation [48]. They included bafilomycin A1, chloroquine, and ammonium chloride (NH_4_Cl). Bafilomycin A1 is an inhibitor of vacuolar-type-H^+^-ATPases, which blocks the budding of ECVs, resulting in the accumulation of internalized molecules in early endosomes [49–52]. When COS7 and HEK293 cells were treated with bafilomycin A1 at 1 μM for 1 hour prior to electrotransfection, the eTE was significantly decreased in both cells (Fig 6).

**Fig 7:**
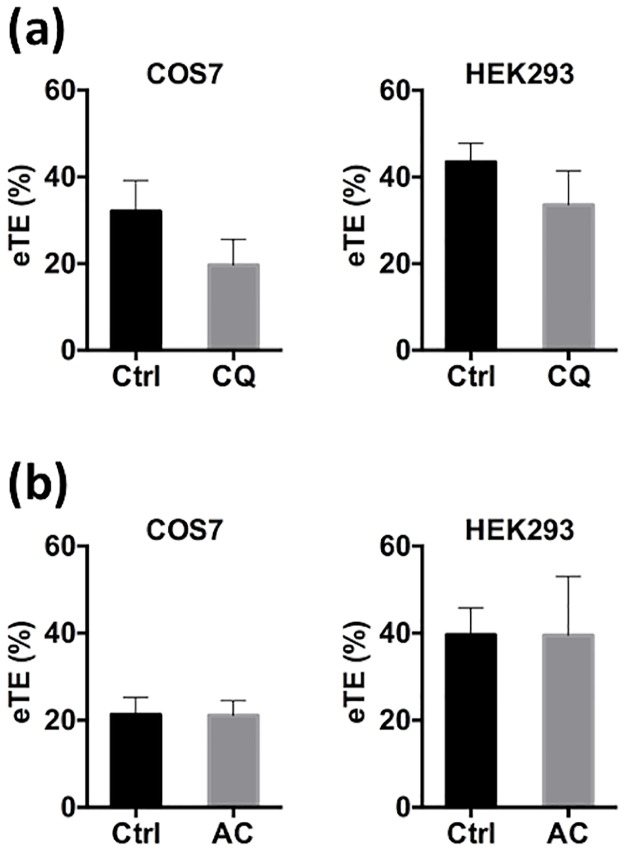
Effects of lysosomotropic agents pretreatment on transgene expression. COS7 and HEK293 cells were treated with either buffer in the control (Ctrl) or a lysosomotropic agent for 4 hours prior to electrotransfection: (a) chloroquine (CQ) at 100 μM, and (b) ammonium chloride (AC) at 10 mM. The treatments had statistically insignificant effects on eTE quantified at 24 hours post electrotransfection. (n = 4, P > 0.05, Mann-Whitney U test).

Chloroquine and ammonium chloride treatments release internalized molecules at the lysosomal stage where the low pH environment in lysosomes leads to protonation of entrapped agents with a high buffering capacity. The protonation causes H^+^, Cl^-^, and water to flow into the lysosomes, resulting in osmotic swelling and eventual rupture of the vesicles [11]. In the current study, we observed that pretreatment of COS7 and HEK293 cells with these lysosomotropic agents, chloroquine (100 μM) and ammonium chloride (10 mM), for 4 hours prior to electrotransfection insignificantly altered eTE (Fig 7a and 7b). The pretreatment was required because the lysosomotropic agents need to be in lysosomes before pDNA reached the lysosomal stage. These data suggested that transient interruption of vesicular transport of pDNA early in the progression of intracellular trafficking in cells had significant effects on gene delivery, and that intracellular trafficking of the naked pDNA which was crucial for gene delivery, might involve routes that excluded lysosomes.

## Discussion

The study showed that eTE could be significantly decreased by PCI-induced endosomal escape at different stages during intracellular transport, or inhibition of endosomal progression by a chemical inhibitor, bafilomycin A1. However, the treatment with lysosomotropic agents, chloroquine and ammonium chloride, had little effects on eTE. These data suggested that vesicular transport was essential for gene delivery via electrotransfection, and that intracellular trafficking of pDNA involved in electrotransfection might exclude routing to lysosomal compartments.

Observations in the study might provide clues on endocytic pathways that were involved in pDNA delivery when they were compared to chemical methods of gene delivery. Cationic polymers and cationic lipids have been widely used in gene delivery. They differ in terms of chemical structures, interactions with nucleic acids, and behaviors within cells, yet their mechanisms of uptake are similar. They all bind to the plasma membrane via nonspecific, electrostatic interactions between the positively charged complex and the negatively charged plasma membrane, and are internalized by cells via endocytosis [17,53]. However, intracellular trafficking of lipoplexes and polyplexes may follow different pathways, presumably because their chemical structures are different [47]. For electrotransfected pDNA, which does not contain any chemical coatings, we expected that its interactions with intracellular structures would be different from those of lipoplexes and polyplexes, and that it is vulnerable to degradation by endonucleases in cells. Since intracellular trafficking of electrotransfected pDNA has been poorly studied in the past, the discussion below is focused on previous studies of non-viral gene delivery, hoping that the discussion may provide some clues on mechanisms of electrotransfection.

### Effects of PCI-induced endosomal escape on non-viral gene delivery

Previous studies have shown that PCI positively affects efficiency of non-viral gene delivery using non-lipid based cationic vectors, such as polyarginine, PLL, and PEI [54,55], where cellular uptake of these vectors was mediated by endocytosis. However, effects of PCI on transfection efficiency can be minimal or become negative when cationic lipid-based vectors were used for gene delivery. For example, PCI-induced endosomal escape resulted in a decrease in transfection efficiency for N-[1-(2,3-dioleoxyloxy)-propyl]-N,N,N-triemethylammonium-methyl-sulfate (DOTAP) and lipofectin [56,57], and had no effect on *β*AE-DMRIE mediated transfection, unless combined with a helper lipid, 1,2-dioleoyl-sn-glycero-3-phosphoethanolamine (DOPE) [54]. Mechanisms of the negative effect of PCI treatment on lipid methods of gene delivery are still unclear. For *β*AE-DMRIE mediated transfection, the lipoplexes may not be able to escape from endosomes after PCI treatment. Thus, it needs DOPE to further destabilize the endosomal membrane [58–60]. Alternatively, the negative effect may be attributable to an inability of pDNA to dissociate from cationic lipids for successful transgene expression in cells [57], or conversely that the cationic lipids do dissociate from pDNA, leaving the naked pDNA vulnerable to endonucleases [61]. More studies need to be performed in the future to test these hypotheses. PCI also negatively affected efficiency of electrotransfection (see Figs 4 and 5). Although mechanisms remain uncertain, we hypothesized that naked pDNA was exposed to endonucleases in cells after light treatment. The treatment permeabilized the endosomal membranes, allowing endonucleases to diffuse into endosomes, and pDNA to escape from endosomes. Both processes could cause rapid degradation of pDNA and thus reduction in gene delivery efficiency.

### Effects of lysosomotropic agents on non-viral gene delivery

Lysosomotropic reagents are often utilized to increase transfection efficiency for gene delivery methods that are limited by the ability to induce endosomal escape prior to enzymatic degradation in the lysosome [62–64]. For example, chloroquine, a lysosomotropic agent, can prevent fusion of endosomes and lysosomes, and the resultant DNA degradation in the acidic environment in lysosomes through inhibition of acidification in late endosomes and lysosomes [11,15,62,63,65]. Chloroquine has been used to increase transfection efficiency for calcium phosphate, DEAE-dextran, and some liposome-mediated gene delivery [63,65–68]. However, effects of lysosomotropic agents on polymer and some lipid based methods of gene delivery are still controversial in the literature. In some studies, chloroquine treatment can positively affect gene delivery by lipofectamine [15] and lipopolyamine [30]. Additionally, it has improved DOTAP and lipofectin-mediated gene delivery, designed specifically with TAT PTD to facilitate endosomal escape [69]. Similar results have also been observed for PLL [70] and lactosylated PLL [71] based methods of gene delivery in HepG2 cells. However, some methods of gene delivery, including cationic polymers and lipids, experience a decrease in efficiency upon chloroquine pretreatment. One such method is PEI-mediated gene delivery [72,73]. A possible explanation for the decrease in efficiency may be related to the inherent ability of PEI to independently induce endosomal escape prior to reaching the lysosomal stage [17], although the exact mechanisms remain unknown. Similarly, treatment of cells with bafilomycin A1 or chloroquine reduced the efficiency of diC14-amidine based methods for gene delivery [47]. Effects of chloroquine treatment on transfection efficiency may depend on lipid formulations. For example, chloroquine treatment (100 μM) decreased transfection efficiency of DMRIE, but increased the efficiency of DORIE. It is worth to note that the two lipids are synthesized similarly with only one minor difference, where 3-dimethylamino-1,2-propanediol was condensed with myristyl mesylate to form DMRIE but oleyl mesylate to form DORIE [74]. Results from the study suggest that small changes in cationic lipid structures can influence outcome of treatment with lysosomotropic reagents. Another interesting observation in the literature is that efficiency of gene delivery by lactosylated PLL was increased after chloroquine treatment but minimally changed after treatment of HepG2 cells with ammonium chloride, another lysosomotropic agent [71]. Ammonium chloride treatment also reduced the efficiency of PLL-mediated gene delivery by 70–90% in COS1 cells [75]. The conflicting findings described above suggest that vector structures and transfection conditions are critical for determining effects of lysosomotropic agents on transfection efficiency.

### Intracellular trafficking after endosomal escape

It is commonly accepted that endosomal escape alone is insufficient for gene delivery because pDNA must travel in the cytosol to reach the nucleus in order to be transcribed [47,76]. For example, it has been shown that cationic lipoplexes injected directly into the nucleus do not result in expression [77]. Previous studies have also shown that upon delivery to the cytoplasm, only cells with plasmids localized in the perinuclear region achieve high levels of transgene expression [78–80]. Furthermore, cytosolic transport of pDNA is hindered by nuclease degradation after escape from endosomes. There is an inverse correlation between the speed with which pDNA travels through the cytosol and the amount of DNA that is degraded [81]. The rate of pDNA degradation varies in different cells [1]. In HeLa and COS cells, the half-life of pDNA is 50-90 min [82]. Unlike other non-viral vectors, electrotransfection delivers naked pDNA into cells, making it more vulnerable to degradation when released into the cytosol. Therefore, metabolic instability of pDNA is particularly a concern for electrotransfection and other physical delivery methods that introduce naked pDNA into cells.

The ability of pDNA to reach the nucleus upon release from endosomes relies also on its capacity to move through the crowded cytoplasm. In fact, DNA fragments larger than 2,000 base-pairs have a negligible diffusion distance in the cytoplasm within a reasonable physiological time frame before being degraded [81], and only 1 out of 1,000 plasmids injected into the cytoplasm can successfully traffic to the nucleus [76,77,83]. These data indicate that it is highly unlikely for naked pDNA to effectively diffuse in the cytoplasm to reach the perinuclear region [84].

### Alternatives to endosomal escape for pDNA delivery to the nucleus

Whether endosomal escape is necessary for pDNA delivery is an interesting question. At present, answers to the question are largely unknown for all non-viral gene delivery methods [47]. Specifically, it remains unclear how pDNA released from endosomes traffics to and enters the nucleus [85,86]. A widely accepted notion is that DNA enters the nucleus via the nuclear pores with a functional diameter of approximately 25 nm, which is smaller than the hydrodynamic diameter of pDNA (~100 nm). Thus, the nuclear entry requires nuclear localization signal to facilitate the transmembrane transport [87]. A second hypothesis is that endosomes containing pDNA interact and fuse with the endoplasmic reticulum, subsequently releasing pDNA into the lumen of the reticulum [47]. This may grant easier access of the pDNA to the nucleus due to the continuous network present between the nuclear and reticulum membranes [47]. A third possibility is specific for lipoplexes that fuse with the nuclear membrane and release the pDNA into the nucleus [88], although it is still unclear how the lipoplexes cross both the outer and inner nuclear membranes to release the pDNA. Finally, it is possible that pDNA can be delivered to the nucleus by fusion of the endosomal compartment directly with the nuclear membrane, and subsequently unloading pDNA into the nucleus. This hypothesis is supported by the identification of nuclear envelope-associated endosomes (NAE), which are early endosomes that localize to the perinuclear region, by several groups studying nuclear delivery of cell surface receptors [89–92], but this mechanism has not yet been observed for pDNA delivery.

In summary, we showed that endosomal escape was discouraged for successful pDNA delivery via electrotransfection. This observation was similar to previous reports of gene delivery with cationic lipid-based vectors. The parallels between lipid-based delivery methods and electrotransfection implied that these two approaches to gene delivery might function by similar mechanisms. Results from the study will enlighten further efforts directed at enhancing the efficiency and safety of electrotransfection for applications in basic research and clinical treatment of diseases.
